# Depicting pseudotime-lagged causality across single-cell trajectories for accurate gene-regulatory inference

**DOI:** 10.1093/pnasnexus/pgad113

**Published:** 2023-03-30

**Authors:** Caleb C Reagor, Nicolas Velez-Angel, A J Hudspeth

**Affiliations:** Howard Hughes Medical Institute and Laboratory of Sensory Neuroscience, The Rockefeller University, New York, NY 10065, USA; Tri-Institutional PhD Program in Computational Biology and Medicine, New York, NY 10065, USA; Present address: 1230 York Avenue, Campus Box 314, New York, NY 10065, USA; Howard Hughes Medical Institute and Laboratory of Sensory Neuroscience, The Rockefeller University, New York, NY 10065, USA; Howard Hughes Medical Institute and Laboratory of Sensory Neuroscience, The Rockefeller University, New York, NY 10065, USA

**Keywords:** gene-regulatory inference, gene-regulatory networks, deep learning, single-cell omics, auditory hair cells

## Abstract

Identifying the causal interactions in gene-regulatory networks requires an accurate understanding of the time-lagged relationships between transcription factors and their target genes. Here we describe DELAY (short for *De*picting *La*gged Causalit*y*), a convolutional neural network for the inference of gene-regulatory relationships across pseudotime-ordered single-cell trajectories. We show that combining supervised deep learning with joint probability matrices of pseudotime-lagged trajectories allows the network to overcome important limitations of ordinary Granger causality-based methods, for example, the inability to infer cyclic relationships such as feedback loops. Our network outperforms several common methods for inferring gene regulation and, when given partial ground-truth labels, predicts novel regulatory networks from single-cell RNA sequencing (scRNA-seq) and single-cell ATAC sequencing (scATAC-seq) data sets. To validate this approach, we used DELAY to identify important genes and modules in the regulatory network of auditory hair cells, as well as likely DNA-binding partners for two hair cell cofactors (Hist1h1c and Ccnd1) and a novel binding sequence for the hair cell-specific transcription factor Fiz1. We provide an easy-to-use implementation of DELAY under an open-source license at https://github.com/calebclayreagor/DELAY.

**Significance Statement**

The sequencing of genes expressed in single cells provides detailed information about the developmental programs that define the identities and states of various cell types, but few computational methods can use the dynamic information encoded in these representations to identify causal mechanisms. By exploiting advances in machine learning, we develop a deep neural network that learns from temporal features of gene regulation to identify direct regulatory interactions between transcription factors and their target genes. Our method provides mechanistic insights into the development and function of cells by generating high-confidence predictions for interactions in complex gene-regulatory networks.

## Introduction

Single-cell sequencing technologies can provide detailed data for the investigation of heterogeneous populations of cells collected at specific times—so-called snapshots—during cellular differentiation or dynamic responses to stimulation (1). However, owing to inherent delays in molecular processes such as transcription and translation, static measurements from individual cells cannot reveal the causal interactions governing cells’ dynamic responses to developmental and environmental cues (2–4). Because population-level heterogeneity in tissues often reflects the asynchronous progression of single cells through time-dependent processes, observed patterns of gene expression can nonetheless indicate the stages of development to which individual cells belong (5). Many algorithms exploit these cell-to-cell differences to infer dynamic trajectories and reconstruct cells’ approximate temporal progressions along inferred lineages in pseudotime (6, 7).

Several methods for gene-regulatory inference rely on pseudotime in Granger causality tests, which try to determine whether new time series can add predictive power to inferred models of gene regulation (8, 9). However, Granger causality-based methods can be error-prone when genes display nonlinear or cyclic interactions or when the sampling rate is uneven or too low (9–12). Because pseudotime trajectories exhibit these problems, Granger causality-based methods often underperform model-free approaches that exploit pure statistical dependencies in gene expression data (9, 13, 14).

By contrast, deep learning-based methods make no assumptions about the temporal relationships or connectivity between genes in complex regulatory networks; instead, these data-driven approaches learn general features of regulatory interactions (15, 16). Here, we describe a deep learning-based method termed *De*picting *La*gged Causalit*y* (DELAY) that learns gene-regulatory interactions from discrete joint probability matrices of paired, pseudotime-lagged gene expression trajectories. Our data suggest that DELAY can address many shortcomings of current Granger causality-based methods and provide a useful, complementary approach to overcome common limitations in the inference of gene-regulatory networks from single-cell data.

## Results

### A convolutional neural network predicts pseudotime-lagged gene-regulatory relationships

**Fig. 1.**
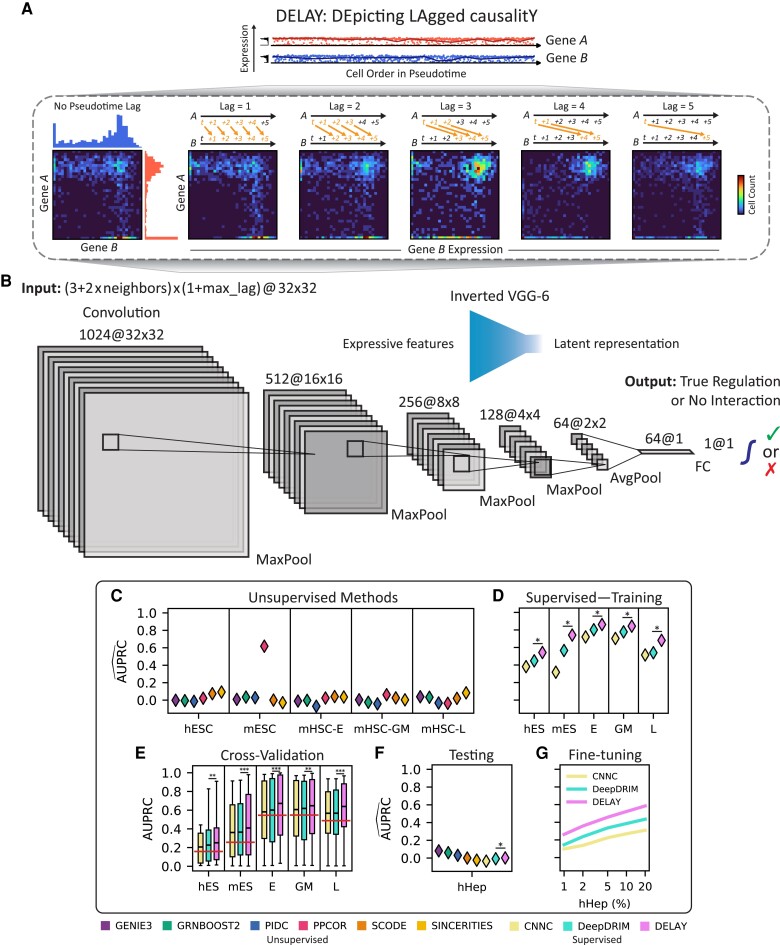
Pseudotime-lagged causality allows accurate inference of gene-regulatory networks. A) Shifting the gene expression trajectory of transcription factor *A* forward in pseudotime with respect to that of target gene *B* generates a series of unique joint probability distributions. The resulting joint probability matrices contain gene coexpression signatures—in the upper-left, upper-right, and lower-right regions—that indicate *A* directly regulates *B*. B) The inverted architecture of DELAY is wide at the beginning of the network and progressively narrows to a one-dimensional vector followed by a linear classifier and sigmoid activation function to generate gene regulation probabilities. The network uses leaky ReLU activations and padded 3 × 3 convolutions throughout. C and D) DELAY outperforms eight of the most popular methods for inferring gene-regulatory interactions across several benchmark scRNA-seq data sets, including six unsupervised methods (C) and two supervised methods (D). E) All three supervised methods perform slightly worse upon cross-validation, but DELAY still outperforms all other methods on average, with the exception of PPCOR for a single metric. F and G) DELAY does not immediately generalize to a new testing data set (F) but outperforms all other methods if fine-tuned on a small percentage of the new data (G). Values for the area under the precision–recall curve (AUPRC) in C), D), F), and G) are normalized by the proportion of positive examples per data set (horizontal lines, E) and averaged across five model replicates for supervised methods. Boxes in E) show the first quartiles, means, and third quartiles for the per- transcription factor AUPRC values across the *k* = 5 validation folds. The statistical significance between DELAY and the next-best neural network was assessed using a one-sided Wilcoxon signed-rank test (*P ≤ 0.05; **P ≤ 0.01; ***P ≤ 0.001).

To predict gene-regulatory relationships from single-cell data, we developed a convolutional neural network based on Granger causality (17). The input to DELAY consisted of stacks of two-dimensional joint probability matrices for pairs of transcription factors, putative target genes, and highly correlated “neighbor” genes (18). We constructed the input matrices by aligning the gene expression trajectories of a transcription factor *A* at several lagged positions in pseudotime relative to a target gene *B* to generate joint probability distributions from two-dimensional histograms of gene coexpression at each lag (Fig. 1A). The value of a given lag indicated the ordinal difference between cells’ rank-ordered positions in pseudotime. Each input matrix consisted of the L2-normalized cell number counts from a two-dimensional gene coexpression histogram with 32 fixed-width bins in each dimension, spanning each gene's minimum and maximum expression values. Although the marginal probability distributions for both *A* and *B* remained essentially unchanged at each lag except for cells lost at the leading and lagging edges of the shifted trajectories, realigning the gene expression in pseudotime altered key features of the resulting joint probability matrices. In other words, causally related genes share important pseudotime-lagged patterns of gene coexpression with nearby cells in single-cell trajectories.

Using ground-truth labels from cell type–specific chromatin immunoprecipitation sequencing (ChIP-seq) data (14), we conducted supervised learning to train our neural network to predict whether *A* directly regulates *B*. This procedure resembled a regression in a Granger causality test (17), in which values of a time series *Y* at timepoints $y_{t}$ are regressed against values from another time series *X* at timepoints $x_{t},x_{t-1},x_{t-2},\ldots ,x_{t-T}$ up to some maximum lag *T* to determine whether any time-lagged values of *X* add explanatory power to *Y*’s autoregressive model. DELAY likewise learned higher weights for gene coexpression matrices at specific pseudotime lags that indicated the true regulatory relationship between genes. After comparing several neural network architectures, we selected a six-layered convolutional network trained on pseudotime-aligned (T=0) and five pseudotime-lagged (T∈{1,2,3,4,5}) gene coexpression matrices to predict direct gene-regulatory relationships (Fig. 1B). We also trained the network on lagged coexpression matrices of two highly correlated neighbor genes per gene, that is, the two transcription factors with the highest cross-correlation with *A* and *B* along the single-cell trajectory. Because the neighbor genes can present stronger alternatives to the primary hypothesis that *A* directly regulates *B*, including these matrices for *A* and *B* versus their highly correlated transcription factors reduced false-positive predictions.

### DELAY outperforms several common methods of gene-regulatory inference

We trained DELAY on gene expression data sets from human embryonic stem cells (hESCs) (19), mouse embryonic stem cells (mESCs) (20), and three lineages of mouse hematopoietic stem cells (mHSCs) (21). We generated separate training data sets for each of the hematopoietic lineages, and all data sets contained at least 400 cells per lineage (Table 1). Each trajectory was oriented according to known experimental timepoints or precursor cell types and lineages, and pseudotime values were inferred separately with Slingshot (6) for each lineage (Fig. S1). We chose these data sets because the cell types and trajectories are well characterized and offer cell type–specific ChIP-seq data to generate ground-truth networks (14). Although the three hematopoietic data sets contained similar numbers of examples of true regulation and no interaction, both of the embryonic data sets were class-imbalanced and contained fewer examples of true regulation. To maximize the network's generalizability, we trained DELAY simultaneously on all five gene expression data sets but validated it on each data set individually. We first generated randomly segregated 70–30% splits of all possible gene pair examples for each data set and then merged the 70% splits to create a combined training data set. In a separate analysis, we also cross-validated DELAY using inductive splits wherein specific transcription factors appeared in only the training or validation splits.

**Table.1.** Summary of the data sets used to train, test, and evaluate the neural network.

Data sets					Networks				
Cell type	TC/S	GEO	Set	Cells	TFs	Targets	Examples	Edges	Density
hESC	TC	GSE75748	Train/Val	758	37 (4)	541	20,017	3,166	15.82%
mESC	TC	GSE98664	Train/Val	421	125 (17)	642	80,250	20,580	25.64%
mHSC-E	S	GSE81682	Train/Val	1,071	33	533	17,589	9,592	54.53%
mHSC-GM	S	GSE81682	Train/Val	889	23	523	12,029	6,587	54.76%
mHSC-L	S	GSE81682	Train/Val	847	16	516	8,256	4,026	48.76%
hHep	TC	GSE81252	Test	425	33 (3)	536	17,688	6,171	34.89%
mOHC (RNA)	TC	GSE137299	Eval	1,563	56	556	1,112*	166*	14.93%*
mOHC (ATAC)	S	GSE157398	Eval	391	48	382	764*	157*	20.55%*

Columns cell type to cells describe the single-cell data sets used to train, test, and evaluate DELAY, and columns TFs to density describe the corresponding gene-regulatory networks. For three of the networks, differentially expressed transcription factors without target genes (in parentheses) were included only as target genes. Network density is equal to the number of edges from the ground-truth ChIP-seq data divided by the total number of examples. E, erythroid lineage; GM, granulocyte–monocyte lineage; L, lymphoid lineage; mOHC, mouse outer hair cell; S, snapshot; TC, time course; TF, transcription factor. *Partial ground-truth network.

After training DELAY on the combined 70% splits, the network outperformed eight of the most popular approaches for inferring gene-regulatory relationships, including six unsupervised methods (8, 22–26 ) (Fig. 1C) and two deep convolutional neural networks (18, 27) (Fig. 1D). We measured the performance of the six unsupervised methods across the combined training and validation splits for each data set and then compared the results with the performance of the supervised methods on the held-out examples alone. With one exception, the deep learning-based methods outperformed all others according to the areas under both the precision–recall (PR) and receiver operating characteristic (ROC) curves (Fig. S2). Moreover, DELAY outperformed all the other methods according to both metrics, even though one of the deep learning-based methods, DeepDRIM, was trained on 5-fold as many neighbor gene matrices. Upon separate cross-validation, DELAY performed slightly worse on inductive splits but with a single exception outperformed all other methods on average (Fig. 1E). Across three of the training data sets, DELAY also outperformed a modified version of DeepDRIM that was trained on the same pseudotime-lagged input matrices as DELAY (Fig. S2). Together, these results suggest that DELAY outperforms other methods because it can better learn important gene-regulatory features from pseudotime-lagged gene coexpression matrices.

### Transfer learning allows DELAY to predict novel gene-regulatory networks from new single-cell data sets

To test whether DELAY generalizes to new data sets, we examined the human hepatocyte (hHep) gene-regulatory network using an additional data set with over 400 single cells and known ground-truth interactions from ChIP-seq data (14, 28). We inferred the network by the previous methods and found that tree- and mutual information-based methods performed slightly better than deep learning-based methods, which were not initially trained on the new data and consequently performed comparably to random predictors (Fig. 1F). To determine whether this lack of generalizability arises from batch effects in the single-cell data, we used transfer learning to fine-tune the three deep learning-based methods on a small number of randomly segregated examples from the new data set while validating on the rest of the examples (Fig. 1G). After training each network on only 1% of the new examples and validating it on the remaining 99%, the networks’ performance matched or exceeded that of the unsupervised methods; training on up to 20% of the new examples yielded further performance increases. DELAY again outperformed every other deep learning-based method. During fine-tuning, we also observed a monotonic increase in the value of the mean validation metric across PR and ROC curves, suggesting that the networks can learn useful features of gene regulation after longer training periods. Moreover, these results demonstrate that DELAY can accurately predict gene-regulatory relationships from new data sets with partially known ground-truth labels.

### DELAY performs well with new input configurations, augmented matrices, and modified data sets

**Fig. 2.**
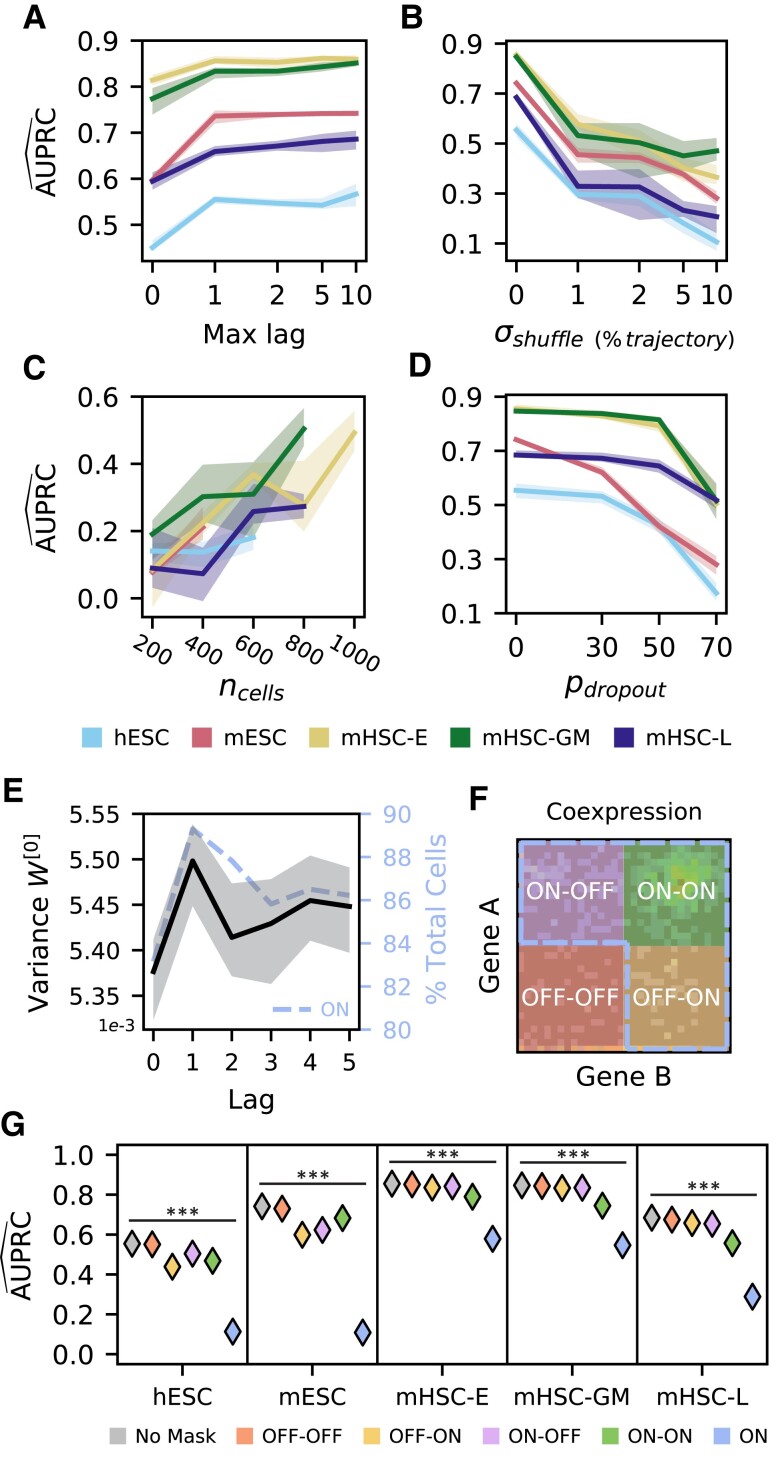
The neural network relies on cell order in pseudotime and gene expression strength. A) Training new models of DELAY on increasing numbers of pseudotime-lagged matrices gives the largest performance increase when using up to a single lag. B) Reordering single cells in pseudotime sharply decreases performance, suggesting that DELAY relies on the specific ordering of adjacent cells in each trajectory. C and D) The network is sensitive to random down-sampling of cells across data sets (C) but relatively more robust to induced, additional gene dropouts in weakly expressing cells (D), suggesting that DELAY relies heavily on highly expressing cells. E and F) The network learns larger input weights for lagged matrices of the first pseudotime lag (E), which also contain more cells in the combined “ON” region ($A_{\mathrm{ON}}\cup \;B_{\mathrm{ON}}$; dotted outline, F) on average across training data sets (dotted line, E). The combined “ON” region is comprised of the upper-left “ON–OFF” quadrant ($A_{\mathrm{ON}}\cap \;B_{\mathrm{OFF}}$), upper-right “ON–ON” quadrant ($A_{\mathrm{ON}}\cap \;B_{\mathrm{ON}}$), and lower-right “OFF–ON” quadrant ($A_{\mathrm{OFF}}\cap \;B_{\mathrm{ON}}$). G) Masking different regions of the input matrices shows that the network relies heavily on the combined “ON” region. The lines and shaded regions in A–E) show the average and full range of values across five model replicates, and the markers in G) show the average values across model replicates. The statistical significance in G) was assessed with a Kruskal–Wallis test (***P ≤ 0.001).

Using different numbers of pseudotime-lagged gene coexpression matrices or neighbor gene matrices, as well as examples from modified data sets with fewer cells or additional gene dropouts, we next examined the performance of DELAY across various input configurations. We employed the original data sets to train new models on gene coexpression matrices of up to 10 pseudotime lags (Fig. 2A) or up to 10 neighbor genes, as well as on gene coexpression matrices with varying dimensions and resolutions (Fig. S3). Training DELAY on at least one pseudotime-lagged matrix or with at least one neighbor gene greatly increased the network's performance across all data sets. Although training DELAY on up to 10 pseudotime-lagged matrices resulted in comparable or slightly better performance across all data sets, training the network on more than two neighbor gene matrices per gene decreased performance in some instances. Adding channel masks for specific lagged matrices suggested that DELAY relies on redundancies across all available lagged inputs (Fig. S3).

We also characterized DELAY's performance on pseudotime-shuffled trajectories and observed a sharp decrease in performance after reordering nearby cells in each trajectory (Fig. 2B). This result suggests that the network relies heavily on the specific ordering of adjacent cells in each trajectory. Upon examining DELAY's performance on modified data sets containing fewer single cells (Fig. 2C) or additional gene-dropout noise (Fig. 2D), we also discovered that the network was more sensitive to down-sampling of single cells than to gene expression losses in low-expressing cells alone. These results indicate that DELAY relies more heavily on highly expressing cells and therefore assigns larger input weights for the first lagged input because on average that input contains stronger features of gene activation than other lags (Fig. 2E and F). To investigate this hypothesis, we used augmented input matrices with masked regions to show that DELAY relies heavily on the combined upper-left, upper-right, and lower-right regions ($A_{\mathrm{ON}}\;\cup \;B_{\mathrm{ON}}$) across all lagged matrices (Fig. 2G). By performing a post hoc analysis of the statistical dispersion across correctly inferred gene pair examples, we additionally found that DELAY generally performs better on transcription factors with stable gene expression along single-cell trajectories (Fig. S3).

### DELAY recognizes causal relationships in hierarchical and cyclic gene-regulatory interactions

**Fig. 3.**
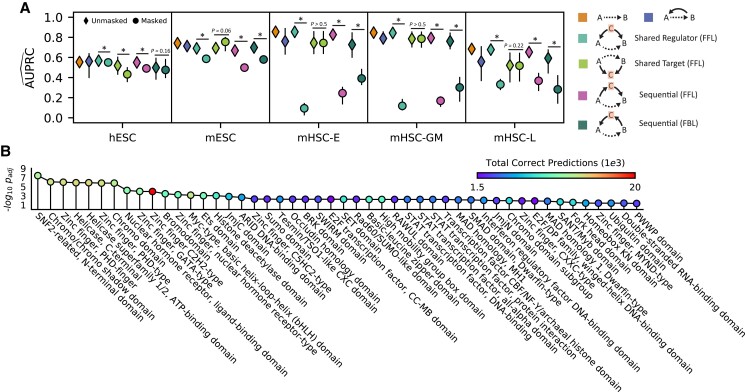
DELAY uncovers causal, cyclic, and context-specific gene-regulatory relationships. A) The network's performance when inferring putative transcription factor-target gene pairs across two- and three-gene motifs in the validation set suggests that DELAY can distinguish between different types of hierarchical and cyclic gene regulation. Upon exclusion of one or more neighbor genes *C* from the input features of the validation examples, the network's performance declines significantly if *C* is a shared or sequential regulator of *A* and *B*, but not if *C* is a shared target. B) GO-term enrichment indicates that DELAY performs best on zinc-finger proteins (PHD-type, GATA-type, C2H2-type, NHR-type, and C5HC2-type), bHLH factors (Myc-type), and other chromatin remodelers (SNF2-related, chromodomain, bromodomain, HDA domain, and helicase). Markers and error bars in A) show the average values and full range of performance across five model replicates. The statistical significance in A) was assessed with a one-sided Wilcoxon signed-rank test (*P ≤ 0.05) and in B) with a Fisher's exact test (*P*_adjusted_ ≤ 0.05).

To further investigate how the selection of neighbor genes can alter DELAY's internal representations and subsequent gene-regulatory inferences, we modified gene pair examples in the original 30% validation splits to exclude all neighbor genes *C* with known interactions across several classes of three-gene motifs. First, we used DELAY to infer gene regulation for potential hierarchical and cyclic interactions across validation set examples (Fig. 3A), including mutual interactions (MIs), feedback loops (FBLs), and three classes of feedforward loops (FFLs). Unlike ordinary Granger causality, DELAY performed well across cyclic interactions including 2-cycles (MIs) and 3-cycles (FBLs), which are ubiquitous among gene-regulatory networks (29). We next excluded from the same validation set examples all input matrices for any neighbor gene of either *A* or *B* known from the ChIP-seq data to be involved in potential FBLs or FFLs, replacing the inputs for all omitted neighbors *C* with the matrices of other highly correlated neighbor genes. Because we saw a significant decrease in performance upon excluding these neighbors *C* in most cases of shared and sequential gene regulation, but not for downstream targets, DELAY apparently recognizes the differences between input matrices of causally related genes and those of merely correlated genes. Moreover, these results suggest that although our network uses principles of Granger causality, it can achieve true causal inference for genes involved in several classes of hierarchical and cyclic regulatory interactions by avoiding several limiting assumptions of ordinary Granger causality.

### DELAY performs best on zinc-finger proteins, bHLH factors, and other chromatin remodelers

To explore how DELAY performs on classes of transcription factors containing different types of DNA-binding domains, we analyzed the enrichment of gene ontology (GO) terms across correctly inferred transcription factors (Fig. 3B). We found that DELAY performs best on zinc-finger proteins, bHLH factors, and other chromatin remodelers. Although DELAY performs well on C2H2-type zinc-finger proteins in terms of the total number of correct predictions, we found that it performs better on other chromatin remodelers and plant homeodomain (PHD) zinc-finger proteins by the overall term enrichment (Table S1). Interestingly, these results indicate that training the network on cell type–specific ChIP-seq data allows the network to identify regulatory relationships involving some non sequence-specific transcription factors and cofactors that nevertheless associate with specific targets at preferred chromatin conformations.

### Predicting the gene-regulatory network of auditory hair cells through multi-omic transfer learning

**Fig. 4.**
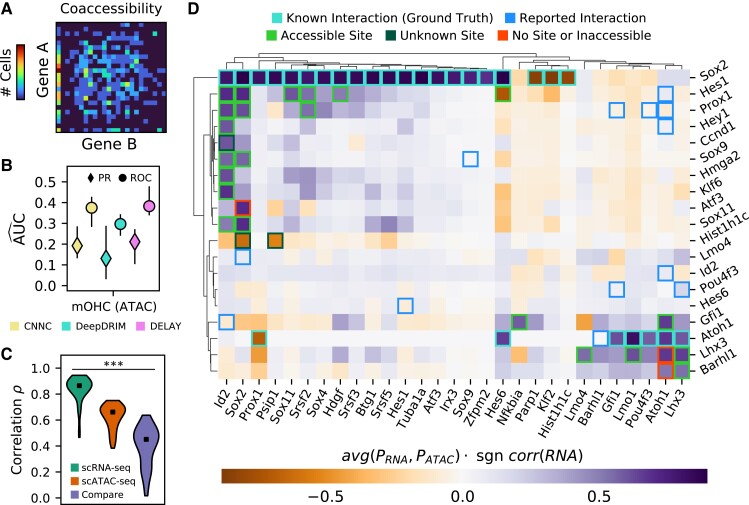
DELAY accurately predicts gene-regulatory interactions in the auditory hair cell network through multi-omic transfer learning. A) An example of an empirical joint probability matrix from scATAC-seq trajectories. B) After fine-tuning the network on lagged scATAC-seq input matrices, DELAY performs comparably with other neural networks and to previous testing on hHep scRNA-Sseq data. C) Training DELAY separately on scRNA-seq and scATAC-seq data sets of hair cell development reveals a stronger correlation between predictions from model replicates than between data sets across the transcription factor-only network. D) Average gene regulation probabilities *P* across the hair cell gene-regulatory network accurately predict interactions between transcription factors (rows) and targets (columns), when comparing known binding sites to open-chromatin peaks in the target genes’ enhancer sequences. Hierarchical clustering with WPGMA reveals distinct gene modules for prosensory genes (*Sox2*, *Id2*, *Hes1*, and *Prox1*) and hair cell genes (*Atoh1*, *Pou4f3*, *Gfi1*, *Lhx3*, and *Barhl1*). Up- or down-regulation was deduced from the correlation in the gene expression data. Markers and error bars in B) show the average values and full range of performance across five model replicates for each neural network. Markers in C) show the median target gene rank correlations across comparisons. The statistical significance in C) was assessed with a Kruskal–Wallis test (***P ≤ 0.001).

We next sought to generate predictions for a cell type with complex but incompletely characterized gene-regulatory dynamics. We devised a pipeline for multi-omic transfer learning to infer the gene-regulatory network for developing mouse outer hair cells, the mechanical amplifiers of the inner ear. We used both gene expression (30) and chromatin accessibility (31) data sets to fine-tune our network. By first calculating the cell-by-gene accessibility scores across annotated genes, we were able to generate lagged input matrices for the single-cell ATAC sequencing (scATAC-seq) data set (Fig. 4A). Because the underlying gene expression and gene accessibility distributions are both zero-inflated (8), the resulting coaccessibility matrices are qualitatively similar to gene coexpression matrices.

To determine whether pseudotime-lagged gene coaccessibility matrices contain features that indicate direct gene-regulatory relationships, we fine-tuned DELAY on the scATAC-seq matrices using ground-truth examples collected from two cell type–specific data sets of Sox2 and Atoh1 target genes (32, 33). Seventy percent of the randomly segregated ground-truth examples were used for fine-tuning, and the remaining 30% were held out for validation (Fig. 4B). DELAY performed slightly worse by the metric of area under the PR curve than by area under the ROC curve, indicating that the network is better at discriminating false positives than selecting true positives. Although DELAY did not outperform all other deep learning-based methods, the network's performance was comparable with previous training and validation on small fractions of the hHep single-cell RNA sequencing (scRNA-seq) data set, which suggests that gene coaccessibility matrices are also useful for inferring direct gene-regulatory interactions.

We separately fine-tuned the network on all available Sox2 and Atoh1 ground-truth examples from an additional gene expression data set for mouse outer hair cells (30). With previously determined hyperparameters for scRNA-seq data, training DELAY on two graphics processing units (GPUs) required 230 ± 1 min (mean ± SD) per model. We then compared the target gene rank correlations between the inferred transcription factor-only gene-regulatory networks to determine whether networks inferred from scRNA-seq data and scATAC-seq data were similar (Fig. 4C). Although we observed stronger correlations between predictions from data set-specific models than between average predictions across data sets, the target gene rank correlations between the two data sets were highly variable, and the predictions of some transcription factors agreed better than others. Reasoning that the inferences with better agreement across data sets constituted the best predictions and highest confidence interactions, we derived the consensus hair cell gene-regulatory network from the average predictions across both data sets. The resulting network consisted of 347 predicted transcription factor–target interactions with gene regulation probabilities >0.5.

### DELAY identifies important genes, interactions, and modules in the hair cell gene-regulatory network

We used hierarchical clustering to group transcription factors and target genes in the transcription factor-only gene-regulatory network for hair cells by similarities in their predicted targets and regulators, respectively (Fig. 4D). This procedure revealed two distinct developmental modules corresponding to prosensory genes such as *Sox2*, *Id2*, *Hes1*, and *Prox1* and hair cell-specific genes such as *Atoh1*, *Pou4f3*, *Gfi1*, *Lhx3*, and *Barhl1*. We sought to validate the predicted interactions by comparing the locations of known transcription factor-binding sites (34, 35) to the locations of open-chromatin peaks (31) within 50 kb of target genes’ transcription start sites. Twenty-two of 28 predicted interactions were confirmed by the accessibility of transcription factor-binding sites. Of the six remaining interactions, three were instances of predicted target-gene regulation by transcriptional cofactors lacking true DNA-binding domains. We additionally identified 13 reported interactions that were not detected by DELAY (31, 36–54).

The most notable feature of the inferred gene-regulatory network for hair cells is that *Sox2* and *Id2*—in addition to their proteins’ well-known roles in regulating target genes and maintaining a prosensory cell fate—are themselves the targets of a wide variety of transcription factors including Sox proteins, homeobox factors, zinc-finger proteins, and Notch effectors. Eleven of the 22 interactions confirmed by our binding-site accessibility analysis represented direct regulation of *Sox2* and *Id2*, including mutually activating interactions between *Sox2* and *Sox9*, *Sox11*, *Prox1*, and *Hes1*, and mutual inhibition with *Hist1h1c*. One other study has suggested that Hes1 directly regulates *Sox2* (55).

Another notable feature of the inferred hair cell network is that the LIM-homeobox transcription factor Lhx3 regulates its own expression as well as that of two other LIM-only transcription factors (Lmo4 and Lmo1). This result implies a role for Lhx3 in maintaining the later expression of these important early *Sox2* inhibitors (31, 47, 49). Other key features of the network include *Sox11* regulation by Hes1, *Nfkbia* regulation by Gfi1, and regulation of the splicing factor gene *Srsf2* by the products of three different prosensory genes. Srsf2 was recently predicted to play a role in the splicing of an important deafness gene in humans (56).

### Discriminative motif analysis of target-gene enhancer sequences enables de novo discovery of DNA-binding motifs

**Fig. 5.**
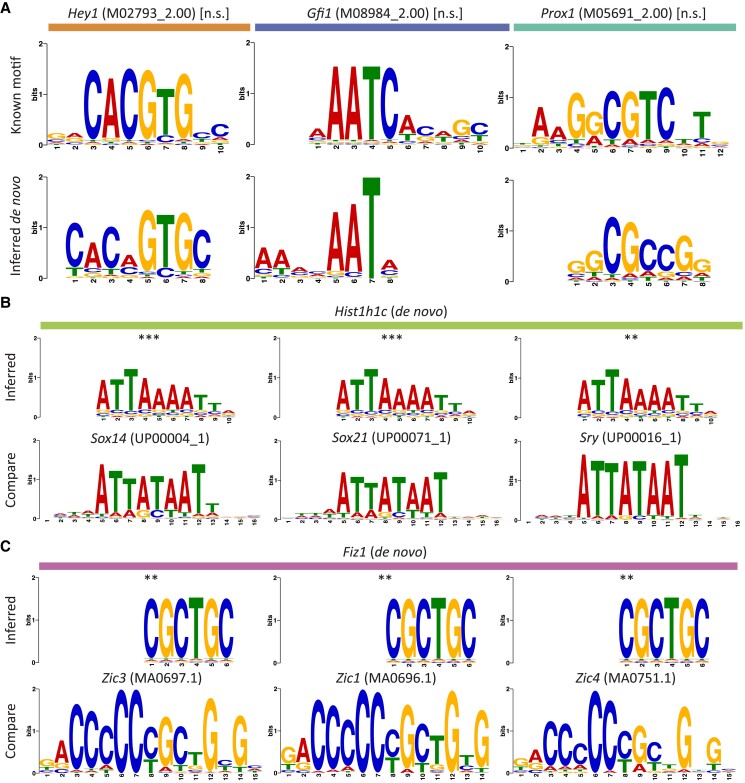
Accurate target-gene predictions enable de novo discovery of DNA-binding motifs. A) Three examples of motifs enriched in the enhancer sequences of predicted target genes (bottom row) closely resemble known DNA-binding motifs (top row) for transcription factors with at least one predicted target gene in the transcription factor-only hair cell gene-regulatory network. B and C) Sequences for putative transcription factors or cofactors may represent novel DNA-binding motifs or indicate sequence-specific interactions through multi-protein complexes. Motifs for two hair cell genes closely match known DNA-binding sequences, suggesting that the linker histone Hist1h1c (B, top row) forms complexes with SoxB2/A factors (B, bottom row) and that the putative C2H2 zinc-finger protein Fiz1 (C, top row) recognizes motifs similar to Zic family C2H2 zinc-finger proteins (C, bottom row). The statistical significance of each motif alignment was estimated using the cumulative density function of all possible comparisons of known motifs across enriched sequences for A) or inferred motifs across all database motifs for B) and C) (**P ≤ 0.01; *** *P* ≤ 0.001).

DELAY permits a complementary approach to typical gene-regulatory inference workflows such as SCENIC (57) that use *cis*-regulatory sequences to identify and discard false-positive interactions resulting from indirect gene regulation (11, 30, 31). Because these methods rely on databases of known DNA-binding motifs, they necessarily overlook predictions for cofactors and putative transcription factors with unknown binding sequences. Instead of using *cis*-regulatory sequences to refine our initial predictions, we introduced enhancer sequence information post hoc to perform discriminative motif analysis within the enhancers of predicted targets in the hair cell network (Figs. 5A and S4). We identified enriched motifs that closely resembled known DNA-binding motifs for nine of 11 transcription factors with at least one predicted target gene in the transcription factor-only hair cell network (Table S2).

We additionally sought to predict the mechanisms by which several identified hair cell cofactors accomplish sequence-specific gene regulation in the absence of true DNA-binding domains. Specifically, we compared sequences enriched in the enhancers of cofactors’ predicted targets to several databases of known DNA-binding motifs to identify the cofactors’ DNA-binding partners (so-called guilt by association). Through this analysis, we were able to determine that the most significantly enriched sequence in the enhancers of the histone Hist1h1c's predicted target genes closely matched known binding motifs for several SoxB2/A transcription factors (Fig. 5B). In addition, a sequence enriched in the enhancers of cyclin Ccnd1's targets accorded with known motifs for the Pbx family of homeobox transcription factors (Fig. S5). These cofactors might form complexes with the identified transcription factors to regulate their target genes in hair cells.

As a final demonstration of DELAY's high-confidence predictions, we used the fine-tuned model for the scRNA-seq data to generate regulatory predictions for transcription factors expressed in at least 20% of developing hair cells (Table S3). After considering these additional transcription factors, we identified the putative C2H2 zinc-finger protein Fiz1 as a likely regulator of *Sox2* and *Hes6*. Discriminative motif analysis of these genes’ enhancer sequences (Fig. 5C) uncovered a likely Fiz1 consensus DNA-binding sequence (5′-CGCTGC-3′) similar to that of other *Sox2* regulators from the Zic family of C_2_H_2_ zinc-finger proteins (58).

## Discussion

Building upon several deep learning-based methods (18, 27), we have demonstrated that combining fully supervised deep learning with joint probability matrices of pseudotime-lagged single-cell trajectories can overcome certain limitations of current Granger causality-based methods of gene-regulatory inference (9, 12), such as their inability to infer cyclic regulatory motifs. Although our convolutional neural network DELAY remains sensitive to the ordering of single cells in pseudotime, the network can nevertheless accurately infer direct gene-regulatory interactions from both time course and snapshot data sets, unlike many supervised methods that rely strongly on the number of available time course samples (15, 16). We suspect that DELAY is sensitive to the specific ordering of adjacent cells in trajectories because pseudotime inference methods such as Slingshot (6) infer lineages from minimum spanning trees that directly depend on cell-to-cell similarities in gene expression values (7).

We have presented a multi-omic paradigm for fine-tuning DELAY on both gene expression and chromatin accessibility data sets for the development of auditory hair cells. The network's accurate predictions allow de novo inferences of known and unknown DNA-binding motifs, establishing DELAY as a complementary approach to common methods of gene-regulatory inference. Our method's high-confidence predictions across the hair cell gene-regulatory network also support it as an attractive option for experimentalists with limited resources to predict true gene-regulatory relationships from complex, large-scale gene-regulatory networks while avoiding spurious, indirect interactions often introduced through unsupervised methods (14).

We have additionally identified with high confidence interactions between cofactors and target genes in the regulatory network of hair cells that methods such as SCENIC (57) would have overlooked. Using “guilt by association,” we predict that the cyclin Ccnd1—a known transcriptional cofactor found at the enhancers of several *Id* genes during retinal development (59)—forms complexes with the Pbx family of homeobox transcription factors, of which at least one is a known target of Prox1 in the inner ear (60). Moreover, we predict that the retina-specific linker histone Hist1h1c (61) forms complexes with the SoxB2 family of transcription factors, which have known antagonistic effects on *Sox2* expression in hair cells (62). Finally, we predict that the putative hair cell-specific C2H2 zinc-finger protein Fiz1—which is also expressed during retinal development (63)—is a regulator of *Sox2* in hair cells and identified its likely DNA-binding sequence in the enhancers of predicted target genes.

Believing that DELAY will be a valuable resource to the community, we have provided an easy-to-use and open-source implementation of the algorithm as well as pre-trained model weights for subsequent fine-tuning on new single-cell data sets and partial ground-truth labels. Unlike other deep learning-based methods, our implementation of DELAY maximizes usability and prioritizes model flexibility. Although we chose to fine-tune DELAY on cell type–specific ChIP-seq data, future studies may choose to fine-tune DELAY on curated interactions from databases or from gain- or loss-of-function experiments, especially in the absence of ChIP-seq data (14). These additional interactions can also supplement smaller ground-truth data sets, such as those of Sox2 and Atoh1 target genes, to mitigate false-negative predictions. We believe that the modest computational cost of training new models of DELAY will prove a worthwhile investment for future investigations into complex gene-regulatory networks.

## Materials and methods

### Preparing single-cell RNA-seq data sets to train and test the convolutional neural network

To train our convolutional neural network, we used scRNA-seq data sets from two time course studies of endodermal specification of hESCs (19) and mESCs (20) and an in vivo snapshot study of erythroid (E), granulocyte–monocyte (GM), and lymphoid (L) specification in mHSCs (21). We additionally employed a data set from a time course study of the differentiation of hHeps from induced pluripotent stem cells to test our network (28). For each of these studies, we collected the normalized gene expression values, pseudotime values, and ground-truth networks from BEELINE’s supplementary data files (14). In BEELINE, Pratapa et al. collected the normalized gene expression values from the original studies or obtained the values themselves by log-transforming the transcripts per kilobase million. Moreover, those authors used Slingshot (6) to infer the pseudotime values separately for each data set, orienting the inferred trajectories by known experimental timepoints or precursor cell types and lineages. Finally, Pratapa et al. selected genes with differential expression across pseudotime by applying generalized additive models (GAMs) to compute gene variances and their associated *P* values. To train DELAY, we utilized their gene variances and corrected *P* values to select differentially expressed transcription factors ($P_{\mathrm{adjusted}}<0.01$) and 500 additional genes with the highest variance for each data set. We also collected the ground-truth labels from BEELINE’s supplementary data files, which contained tables of transcription factor–target gene interactions curated from cell type–specific ChIP-seq experiments in the ENCODE (64), ChIP-Atlas (65), and ESCAPE (66) databases. Table 1 provides details for each data set including descriptions of the ground-truth networks.

We also characterized our network on modified data sets with shuffled pseudotime, fewer cells, and additional gene-dropout noise. We shuffled pseudotime values across single-cell trajectories using np.random.normal in NumPy (v1.20.2) to select the indices of single cells either leading or lagging successive cells in each trajectory at some length scale σ before swapping the cells’ positions in pseudotime. We also used np.random.choice to select the indices of single cells to retain when down-sampling the number of cells in a data set. Lastly, we induced additional gene-dropout noise in data sets by setting the gene expression values to 0 with a probability of *p* for the bottom *p* percent of cells expressing each gene in a given data set.

### Generating examples of transcription factor–target gene pairs from single-cell data sets

For each data set, we generated mini-batches of 512 transcription factor–target gene pair examples by first enumerating all possible combinations of differentially expressed transcription factors and potential target genes—including both transcription factors and highly varying genes. We then generated aligned and pseudotime-lagged gene coexpression matrices for up to five lags with the following configurations as separate input channels: transcription factor–target, transcription factor–transcription factor, target–target, transcription factor–neighbor (for two neighbors), and target–neighbor (for two neighbors). We concatenated the input channels for each example to form three-dimensional stacks of input matrices with dimensions 42×32×32 (channels × height × width). For each gene, we used np.correlate in NumPy to select the transcription factors with the highest absolute cross-correlation at a maximum offset of five pseudotime lags to use as neighbor genes. We trained five model replicates on unique, randomly segregated 70–30% splits of all possible gene pair examples generated with the random_split function in PyTorch (v1.8.1). Each random split achieved a strict segregation of unique gene pair examples into either the training or validation sets, though gene pairs for the same transcription factor but different target genes appeared in both sets. For separate model cross-validation, we generated five inductive splits across each data set wherein specific transcription factors appeared in only the training (80%) or validation (20%) splits. For both analyses, training splits from individual data sets were subsequently merged to create combined training data sets.

We later used the original randomly segregated validation splits to characterize our network on augmented coexpression matrices and matrices generated from modified single-cell data sets. To augment gene coexpression matrices, we masked with zeros either specific region across all input channels or entire input channels corresponding to specific pseudotime lags.

### Constructing a convolutional neural network to classify lagged gene coexpression matrices

We designed a convolutional neural network based on an inverted VGGnet (67) that uses five convolutional layers to first expand the input to 1,024 channels and then successively halve the number of channels to 64 before classifying examples as either true regulation or no interaction with a fully connected linear layer. Each convolutional layer sums over the two-dimensional cross-correlations (⋆) of 3×3 kernels and input channels *i* to find the features for a given output channel *j*, as shown in Equation 1:


(1) $$\begin{array}{c} \mathrm{output}\;(N_{k},\;C_{\mathrm{ou}t_{j}})\;=\;\mathrm{bias}\;(C_{\mathrm{ou}t_{j}})\;+\overset{C_{\mathrm{in}}}{\underset{i=1}{\sum }}\;\mathrm{weight}s_{\;3\times 3}\;(C_{in_{i}},\;C_{\mathrm{ou}t_{j}})\;⋆\;\mathrm{input}\;(N_{k},\;C_{in_{i}}), \end{array}$$

in which the weights and bias are trainable parameters, *N* is the mini-batch size, *C* is the number of channels, $C_{\mathrm{out}}=C_{\mathrm{in}}/2$ for layers 2 through 5, and all convolutions are zero-padded at the edges of matrices. To preserve the gradient flow during training, we used leaky rectified linear units (ReLUs) with a negative slope of 0.2 (Equation 2) as our nonlinear activation function after each convolutional layer:


(2) $$\begin{array}{c} \mathrm{Leaky}\;\mathrm{ReLU}\;(x)\;=\;\max\;(0,\;x)\;+\;0.2*\min\;(0,\;x). \end{array}$$

As shown in Equation 3, we additionally used 2×2, unpadded max-pooling layers between convolutional layers to identify important features and down-sample activation maps:


(3) $$\begin{array}{c} \mathrm{output}\left(N_{k},\;C_{\mathrm{ou}t_{j}},\;\frac{h_{\mathrm{in}}}{2},\;\frac{w_{\mathrm{in}}}{2}\right)=\;\underset{2\times 2}{\mathrm{stride}}\;\left[\underset{m,n\;\in \;\{0,1\}}{\max}\;\mathrm{input}\;(N_{k},\;C_{in_{j}},\;x_{m,n})\right], \end{array}$$

in which *x* represents the 2×2 input windows and h,w are the height and width of the input channel *j*. After the final convolutional layer, we used global-average pooling (Equation 4) to reduce the remaining 64 feature maps *j* to a single, 64-dimensional vector *x*:


(4) $$\begin{array}{c} \mathrm{output}\;(N_{k},\;C_{\mathrm{ou}t_{j}})\;=\underset{h_{\mathrm{in}}\times w_{\mathrm{in}}}{\mathrm{avg}\;}\;\mathrm{input}\;(N_{k},\;C_{in_{j}}). \end{array}$$

We lastly used a fully connected linear layer (Equation 5) with a sigmoid activation function (Equation 6) to generate gene regulation probabilities:


(5) $$\begin{array}{c} \mathrm{output}\;(N_{k})\;=\;\mathrm{bia}s_{\;\mathrm{FC}}\;+\overset{64}{\underset{i=1}{\sum }}\;\mathrm{weight}s_{\;\mathrm{FC}\;}(x_{i})*\mathrm{input}\;(N_{k},\;x_{i}), \end{array}$$

(6) $$\begin{array}{c} \mathrm{Sigmoid}\;(x)\;=\;\frac{1}{1\;+\;\exp(-x)}, \end{array}$$

where a probability of P>0.5 indicates a true gene-regulatory interaction for the given gene pair example.

### Training and fine-tuning the network on pseudotime-lagged gene coexpression matrices

We used PyTorch's implementation of stochastic gradient descent (SGD) to optimize our network with respect to the class-weighted binary cross-entropy loss, summed across each mini-batch and scaled by the overall batch size 512, as shown in Equation 7:


(7) $$\begin{array}{c} L(y,\;\hat{y})\;=\;-\frac{w_{N}}{512}\overset{N}{\underset{n=1}{\sum }}\;y_{n}*\log\;{\hat{y}}_{n}\;+\;(1\;-\;y_{n})*\log\;(1\;-\;{\hat{y}}_{n}), \end{array}$$

in which *y* and $\hat{y}$ are the target and predicted values (respectively), $w_{N}$ is the fraction of true examples in the mini-batch, and *N* is the size of the current mini-batch (which might be <512). Prior to optimization, we used He initialization (68) with uniform priors to set the weights for all convolutional and linear layers. We used a learning rate (LR) of 0.5 to train each model for up to 100 epochs, validating performance after each epoch and stopping training after 10 or more epochs without an improvement in the mean validation metric across PR and ROC curves. For fine-tuning, we trained each model for up to a maximum of 5,000 additional epochs due to the observed monotonic increase in the value of the mean validation metric. We occasionally reduced the LR to 0.25 or 0.1 if the training became unstable, and we tried several mini-batch sizes ≥24 for fine-tuning and separate model cross-validation. All training and testing was performed on two Nvidia RTX 8000 GPUs.

### Comparing DELAY to other top-performing gene-regulatory inference methods

We compared the performance of our neural network to two other convolutional neural networks, as well as the top six best-performing methods identified in a previous benchmarking study. Using BEELINE, we inferred the gene-regulatory networks for all training and testing data sets with the tree-based methods GENIE3 (22) and GRNBoost2 (23), the mutual information-based method PIDC (24), and the partial correlation and regression-based methods PPCOR (25), SCODE (26), and SINCERITIES (8). We utilized the best parameter values identified in BEELINE for the partial correlation and regression-based methods. For the two deep learning-based methods [CNNC (27) and DeepDRIM (18)], we used the original studies to reconstruct the neural networks in PyTorch before training models on the same randomly segregated training examples as DELAY. In addition to training both neural networks on pseudotime-aligned gene coexpression matrices for primary transcription factor–target gene pairs, we also trained DeepDRIM on 10 neighbor gene matrices per gene as in the original study. Moreover, we separately trained DeepDRIM on five pseudotime-lagged matrices per gene pair with 10 neighbors across the same inductive splits as DELAY to compare the cross-validated performance between the networks.

### Analyzing enrichment of GO terms across correctly inferred gene pair examples

We used Enrichr (69) (https://maayanlab.cloud/Enrichr/) to analyze GO-term enrichment across correctly inferred transcription factors for terms related to InterPro DNA-binding domains (70). We used a Fisher's exact test with Benjamini–Hochberg correction for multiple-hypothesis testing ($P_{\mathrm{adjusted}}<0.05$) to assess the statistical significance for each GO term.

### Preparing single-cell multi-omics data sets to infer the hair cell gene-regulatory network

We used two single-cell data sets from developing mouse outer hair cells (30, 31) to predict the consensus hair cell gene-regulatory network. First, we collected the normalized gene expression values from the original scRNA-seq study (30) and then used reciprocal PCA to integrate single cells across four timepoints of sensory epithelium development into a single data set in Seurat (71) (v3.1.4). Then, we used Slingshot (6) (v1.0.0) to infer pseudotime values across the outer hair cell trajectory and applied LOESS regressions and GAMs to select the genes that were differentially expressed across both the sensory epithelium and pseudotime, respectively. We again selected all differentially expressed transcription factors (P<0.01 after Bonferroni correction for multiple-hypothesis testing) and 500 additional genes with the highest variance and used the integrated gene expression assay in Seurat to generate the corresponding gene coexpression matrices. In a separate analysis, we broadened our selection criteria to encompass all genes that were differentially expressed in at least 20% of the outer hair cells, regardless of their expression across the full sensory epithelium.

For the scATAC-seq data set, we first collected the cell-by-peak chromatin accessibility data from the original study (31). Then, we used the function createGmatFromMat in SnapATAC (72) (v1.0.0) to calculate the cell-by-gene accessibility scores as the counts of each 5 kb bin per gene in the UCSC mouse genome mm10 (73) (TxDb.Mmusculus.UCSC.mm10.knownGene; v3.4.4) that contained at least one open-chromatin peak for a given cell. These values were then log-normalized. We again used Slingshot to infer pseudotime values across the outer hair cell trajectory and applied GAMs to the raw accessibility counts to select the differentially accessible genes across pseudotime. For the inferred scATAC-seq network, we selected all differentially accessible transcription factors and variable genes ($P_{\mathrm{adjusted}}<0.01$) that were also differentially expressed along the scRNA-seq trajectory. Because the scATAC-seq data set had fewer cells than the scRNA-seq data set, we used 24 fixed-width bins in each dimension to generate the corresponding gene coaccessibility matrices prior to fine-tuning.

### Discriminative motif analysis to discover de novo transcription factor-binding motifs

We used the UCSC mouse genome mm10 and twoBitToFa to download the 100 kb enhancer sequences spanning 50 kb upstream and downstream of all target genes’ transcription start sites in the transcription factor-only hair cell gene-regulatory network and then divided them into 100 bp fragments and sorted the resulting sequences into primary (predicted targets) and control (predicted no interaction) groups for each transcription factor with at least one target gene in the network. We then used STREME (74) (v5.4.1) to perform discriminative motif analysis with a *P* value threshold of 0.05 to identify enriched motifs in predicted target genes’ enhancers, which we then compared with either the transcription factors’ known binding motifs from CIS-BP (34), or to all motifs from the JASPAR (75), UniPROBE (76) (mouse), and Jolma et al. (77) databases using TOMTOM (78) (v5.4.1) with an *E* value threshold of 10 for significant alignments.

## Supplementary Material

pgad113_Supplementary_Data

## Data Availability

The processed experimental files for all single-cell data sets used in this study are available on Zenodo at https://doi.org/10.5281/zenodo.7474099; Table 1 lists the Gene Expression Omnibus (GEO) accession numbers for each data set. The saved model weights for DELAY are available on Zenodo at https://doi.org/10.5281/zenodo.7474115. All experimental logs from this study are available at https://tensorboard.dev/experiment/RBVBetLMRDiEvO7sBl452A. We have provided an open-source implementation of DELAY in PyTorch with listed requirements and documentation at https://github.com/calebclayreagor/DELAY.
