# Supplementary material for: Depicting pseudotime-lagged causality across single-cell trajectories for accurate gene-regulatory inference
Source: PNAS Nexus. 2023 Mar 30;2(4):pgad113. doi: 10.1093/pnasnexus/pgad113 (PMC10129065; doi:10.1093/pnasnexus/pgad113)
Supplement: pgad113_Supplementary_Data [file pgad113_supplementary_data.zip › PNASNEXUS-PNASNEXUS-2022-00967-T-s01.pdf]

Supplementary Figures

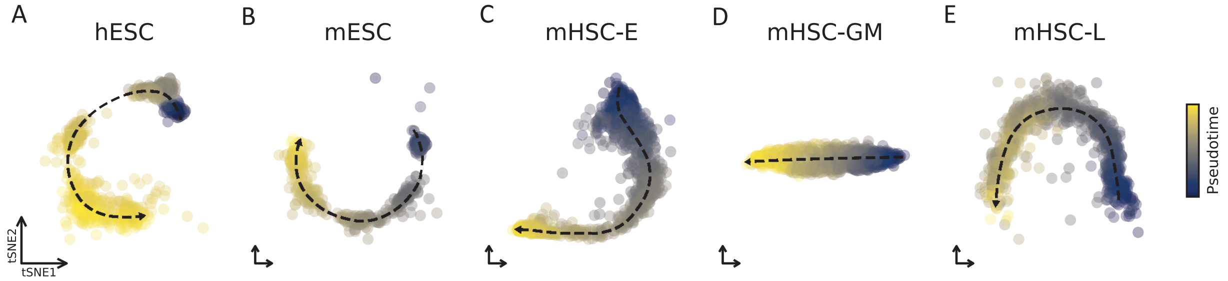

Figure S1: Inferred pseudotime trajectories for the five training datasets. Two-dimensional embeddings of the trajectories for human embryonic stem cells (A), mouse embryonic stem cells (B), and mouse hematopoietic stem cells (C-E) were generated from the differentially expressed genes identified in BEELINE and colored by the inferred Slingshot pseudotime values.

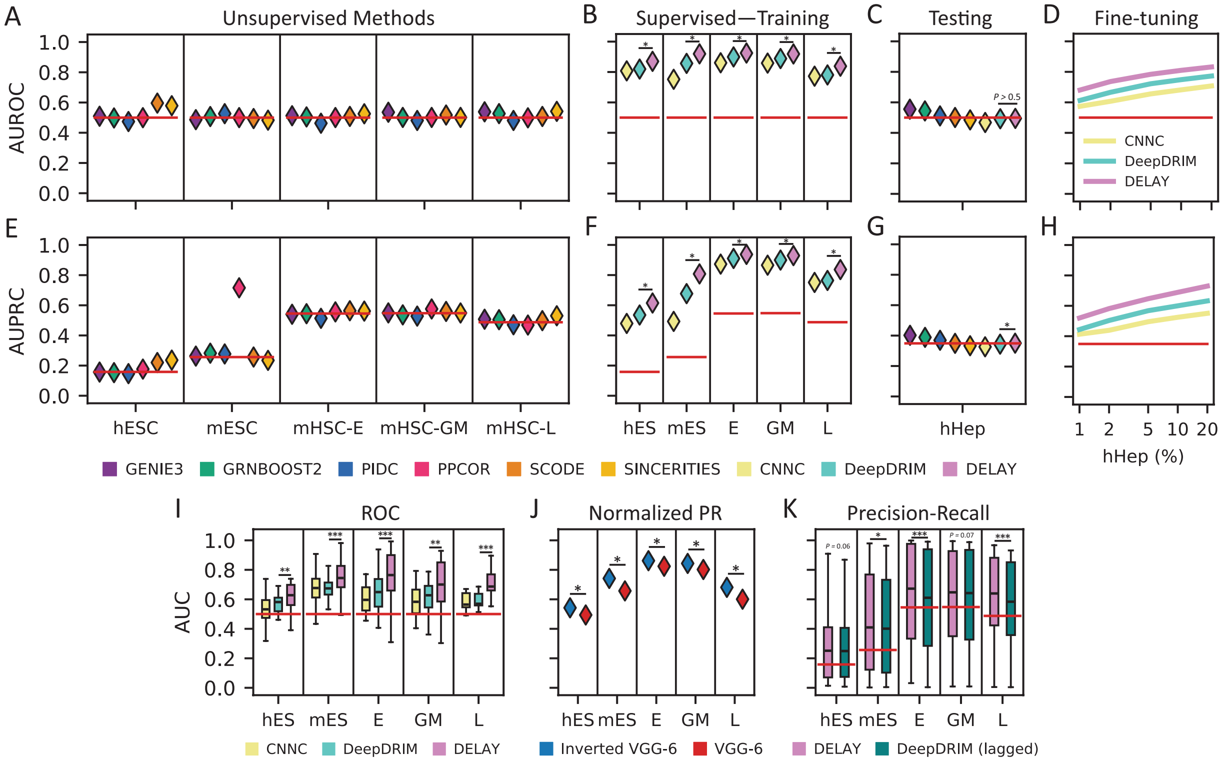

Figure S2: DELAY outperforms unsupervised and supervised methods. (A-H) Unnormalized values for the area under the precision-recall curve (AUPRC) and receiver operating characteristic (AUROC) for DELAY *versus* six unsupervised methods (A, E) and two supervised methods (B, F), with additional testing (C, G) and fine-tuning of supervised methods (D, H). (I) All three supervised methods perform slightly worse by the AUROC metric upon cross-validation, but DELAY still outperforms all other methods on average. (J, K) When trained on the same lagged input matrices, DELAY's inverted VGG-6 network outperforms both a conventional VGG-6 (J) and a modified version of DeepDRIM with lagged inputs (K). Values for the area under the precision-recall (PR) and receiver operating characteristic (ROC) curves are averaged across five model replicates for supervised methods in (A-H) and (J), and normalized by the proportion of positive examples per dataset in (J). Horizontal red lines show the baseline values of either 0.5 in (A-D) and (I) or the proportion of positive examples per dataset in (E-H) and (K). Boxes in (I, K) show the first quartiles, means, and third quartiles for the per-TF AUC values across the k=5 validation folds. The statistical significance in (B, C, F, G) and (I-K) was assessed using a one-sided Wilcoxon signed-rank test (*, $P \leq 0.05$; **, $P \leq 0.01$; ***, $P \leq 0.001$).

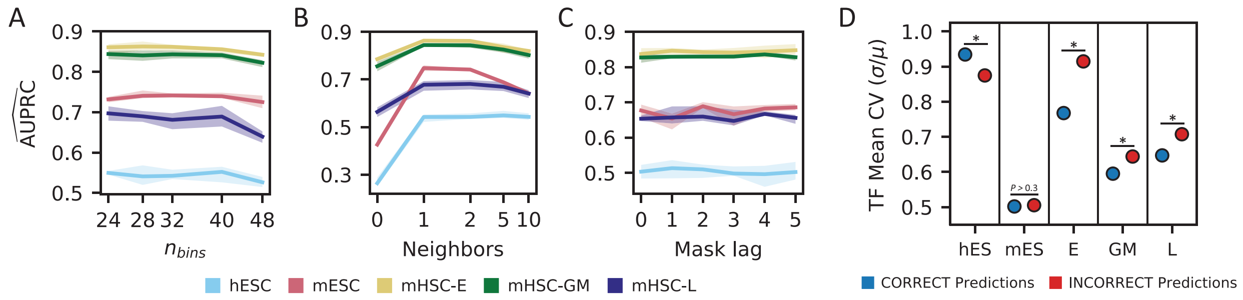

Figure S3: Performance of DELAY across various input configurations. (A) DELAY's performance is robust across input-matrix sizes from 24×24 to 48×48. (B) Training DELAY on up to two neighbor-gene matrices increases performance across all datasets. (C) Testing on datasets with channel masks added to lag-specific input channels suggests that DELAY relies equally on input matrices from each pseudotime lag. (D) Transcription factors (TFs) in correctly inferred gene-pair examples have on average lower coefficients of variation (CVs) for three of five training datasets, suggesting DELAY generally performs best on TFs with stable gene expression. The lines and shaded regions in (A-C) show the average and full range of values across five model replicates, and the markers in (D) show the average values across predictions from model replicates. The statistical significance in (D) was assessed using a one-sided Wilcoxon signed-rank test (*, $P \leq 0.05$).

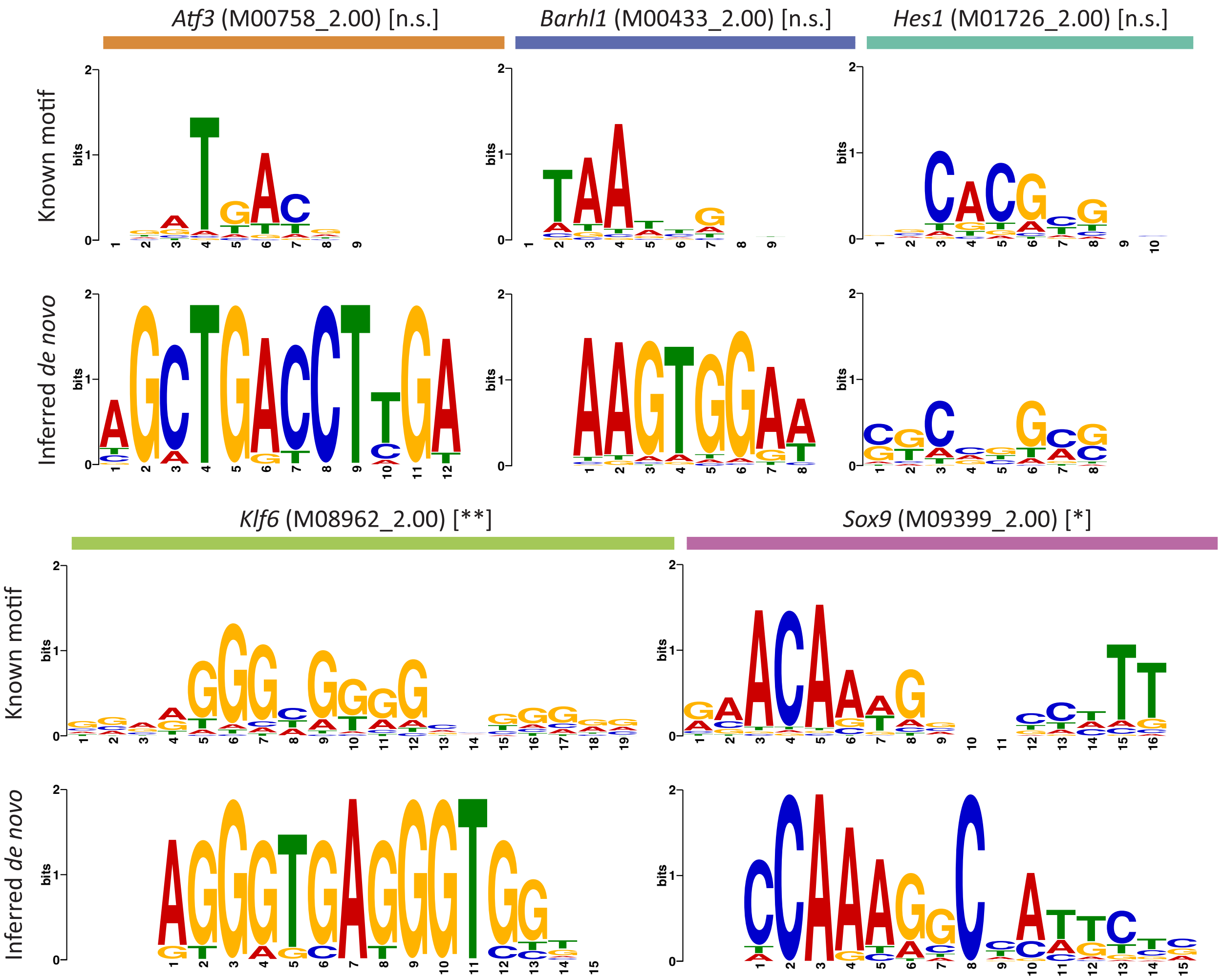

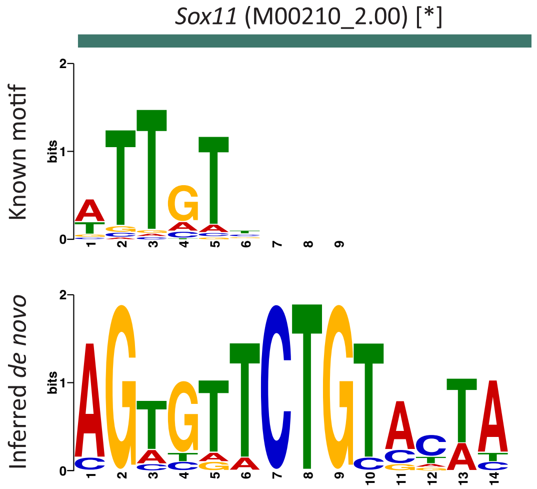

Figure S4: Discriminative motif analysis corroborates gene-regulatory predictions. Six additional examples of enriched motifs in the enhancer sequences of predicted target genes closely resemble known DNA-binding motifs for transcription factors with at least one predicted target gene in the transcription factor-only hair cell gene-regulatory network. The statistical significance of each motif alignment was estimated using the cumulative density function of all possible comparisons of known motifs across enriched sequences (*, $P \leq 0.05$; **, $P \leq 0.01$).

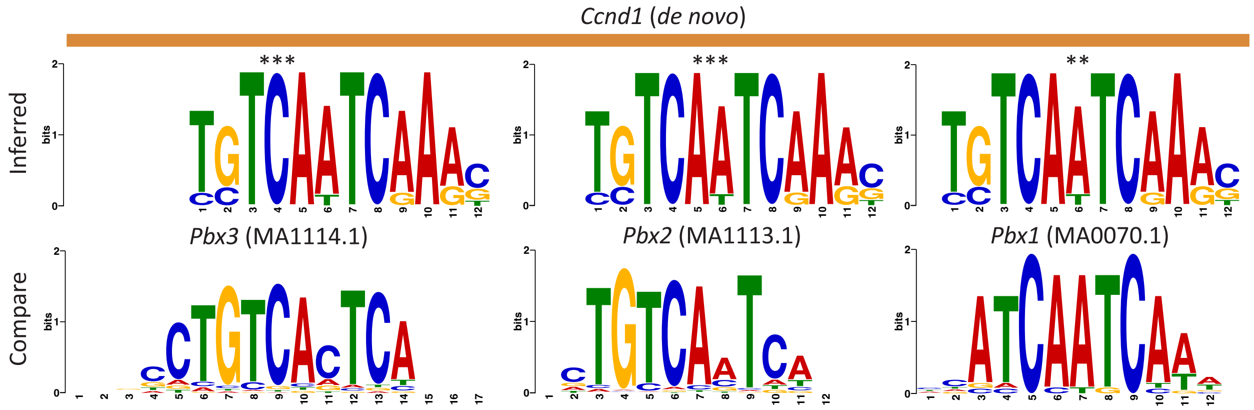

Figure S5: An enriched motif in the enhancers of Ccnd1's predicted target genes suggests a mechanism for cofactor-target interactions. An enriched motif in the enhancer sequences of cyclin Ccnd1's predicted target genes (top row) suggests that the putative cofactor forms complexes with Pbx-family homeobox transcription factors (bottom row) to regulate its target genes in a sequence-specific manner. The statistical significance of each motif alignment was estimated using the cumulative density function of all possible comparisons of the inferred motif across all database motifs (**, $P \leq 0.01$; ***, $P \leq 0.001$).
